# Cough in adult cystic fibrosis: diagnosis and response to fundoplication

**DOI:** 10.1186/1745-9974-5-1

**Published:** 2009-01-18

**Authors:** Hosnieh Fathi, Tanya Moon, Jo Donaldson, Warren Jackson, Peter Sedman, Alyn H Morice

**Affiliations:** 1 Cardiovascular and Respiratory Studies, Hull York Medical School, University of Hull, Castle Hill Hospital, Castle Road, Cottingham, East Yorkshire, HU16 5JQ, UK; 2 Gastro-Intestinal Physiology Laboratory, Castle Hill Hospital, Cottingham, East Yorkshire, HU16 5JQ, UK; 3 Dietetics Department, Castle Hill Hospital, Castle Road, Cottingham, East Yorkshire, HU16 5JQ, UK; 4 Division of Upper Gastrointestinal and Minimally Invasive Surgery, Department of Surgery, Hull Royal Infirmary, Anlaby Road, Hull, HU3 2JZ, UK

## Abstract

**Background:**

Gastroesophageal reflux is one of the most common causes of chronic cough in the general population. Reflux occurs frequently in patients with cystic fibrosis (CF). We undertook laparoscopic Nissen fundoplication in adult CF patients with a clinical diagnosis of reflux cough who had failed conventional medical therapies.

**Objective:**

We determined the response to the surgical route in the treatment of intractable reflux cough in CF.

**Method:**

Patients with refractory cough were assessed by 24 h pH monitoring and oesophageal manometry. Pre-and post-operation cough, lung function and exacerbation frequency were compared. Cough was assessed by the Leicester Cough Questionnaire (LCQ), lung function by spirometry and exacerbation frequency was defined by comparing the postoperative epoch with a similar preoperatively.

**Results:**

Significant abnormalities of oesophageal function were seen in all patients studied. 6 patients (2 females), with the mean age of 34.5 years consented to surgery. Their mean number of reflux episodes was 144.4, mean DeMeester score was 39.2, and mean lower oesophageal sphincter pressure 12.4 mmHg. There was a small change in the FEV1 from 1.03 L to 1.17 (*P *= 0.04), and FVC improved from 2.62 to 2.87 (*P *= 0.05). Fundoplication lead to a marked fall in cough with the total LCQ score increasing from 11.9 to 18.3 (*P *= 0.01). Exacerbation events were reduced by 50% post operatively.

**Conclusion:**

Whilst there is an obvious attention to respiratory causes of cough in CF, reflux is also a common cause. Fundoplication is highly effective in the control of reflux cough in CF. Significant reduction in exacerbation frequency may indicate that reflux with possible aspiration is a major unrecognised contributor to airway disease.

## Background

Cystic fibrosis (CF) is a multisystem disease which impacts the digestive system, sweat glands, and the reproductive tract, but progressive pulmonary insufficiency continues to be major cause of morbidity and mortality.[1] The main respiratory manifestations include chronic bacterial colonisation, cough, bronchiectasis, haemoptysis, emphysema, and pneumothorax. As the disease progresses chronic cough becomes a universal symptom, reported by virtually all patients.[2]

Gastroesophageal reflux, which is increasingly recognised as one of the most common causes of chronic cough in general population, occurs frequently in patients with CF.[3] About one in five newly diagnosed infants with cystic fibrosis have pathological reflux,[4] and a higher frequency (25–55%) has been previously reported in children over 1 year old.[5,6] Similarly, high rates of reflux symptoms, diminished lower oesophageal sphincter pressure and acid reflux are reported in adult CF patients.[7] However, because of the understandable focus on airway disease in CF, reflux as a potential aetiology of chronic cough is often unconsidered. Fortunately, the symptomatology of reflux induced cough allows the clinician to identify cough of gastroesophageal origin.[8]

The medical treatment of oesophageal reflux causing cough is challenging, particularly in the presence of non-acid reflux. In contrast to the treatment of gastroesophageal reflux disease (GORD) causing heart burn, where acid is a vital component, patients with chronic cough frequently fail to respond to full acid suppression with proton pump inhibitors (PPI) and H2 blockers. In the treatment of resistant chronic cough, Nissen fundoplication has been shown to be an effective tool.[9]

We hypothesised that in CF patients with reflux cough not responding to maximal medical therapy, laparoscopic Nissen fundoplication (LNF) could be an alternative approach. Maximal medical treatment was defined as therapeutic trials of the medication listed in table 1, based on the tolerance and compliance of each patient.

**Table 1:** Medical treatment

**Class of drug**	**Duration of Therapy**
Proton pump inhibitor BD + H2-receptor antagonists (Ranitidine) nocte	At least 2 months
Dopamine receptor antagonist (Metoclopramide, Domperidone) TDS	1 month
Cough Suppressant (Morphine, Disofrol)	2 months
GABA agonist (Baclofen) TDS	2 months

## Method

### Patients

30 adult patients with CF diagnosed on the basis of clinical presentations and confirmed by sweat tests or genetic investigation, are under the care of the Hull adult CF unit. 18 patients were considered to have symptoms of reflux cough based on a semi-structured questionnaire, (Appendix 1) [10] and prescribed standard medical treatment. We report our experience in 6 patients who consented to undergo LNF following the failure of maximal medical therapy to control reflux symptoms, particularly chronic cough.

For this study ethical approval was waived by the ethics committee of Hull and East Riding, UK. (Letter dated 19^th ^of June 2008)

### Pre-operative assessment

Oesophageal motility was assessed by solid-state manometry. Ambulatory 24 hr oesophageal pH was used to assess the presence of gastroesophageal reflux. Intra oesophageal pH was measured at a level of 5 cm above the pre determined (via oesophageal manometry) upper border of the lower oesophageal sphincter (LOS),[11] and presented as DeMeester score[12]

### Intervention

Laparoscopic fundoplication was performed in a standard fashion under general anaesthesia with full muscle relaxation using a five port technique. In every case the oesophageal hiatus was fully dissected and the oesophagus mobilised. At least one non absorbable suture was placed to approximate the crura posterior to the oesophagus and to minimise the risk of post-operative herniation. In cases where there was a large pre-existing hiatal defect, additional posterior crural sutures were placed as required. In most cases there was no obvious hiatus hernia demonstrated at surgery. Calibration of the oesophageal hiatus was clinical but in cases of doubt a 56 Fr endoluminal bougie was available to calibrate the appropriate size.

### Outcomes

Our primary outcome measure was the change in cough as assessed by self-administered Leicester Cough Questionnaire (LCQ). Pre- and post-operative changes in spirometry and exacerbation rate as defined by an event requiring antibiotics (oral or IV), were secondary endpoints. Results are expressed as means and standard deviations (SD), and the comparisons pre and post- operatively were made by paired t-test with 95% confidence interval (CI), using SPSS software version 13.

## Results

### Demeester score, esophageal ph and manometry studies pre-operation

Of 18 patients with clinically diagnosed reflux cough, four men and two women with a mean age of 34.5 ± 14.7 years, failed to show any improvement with the standard medical therapy of reflux cough. In the 5 patients consenting to oesophageal studies, the mean number of reflux episodes was 144.4 (range from 97 to 178), and pre-operative mean DeMeester score was 39.2 (± 24). The mean resting pressure of lower oesophageal sphincter was 12.4. Reported cough was highly associated with reflux episodes. (Table 2)

**Table 2:** Demographics and pre-operation assessments

Age	Sex	FVC	FEV1	LOS Resting Pressure 15–30 mmHg	% of time pH > 4	Reflux Episodes NL < 50	DeMeester Score NL < or = 14.7
49	M	1.08	3.20	6.00	15.20	155.00	78.10
27	F	0.77	1.89	9.00	5.90	160.00	29.90
57	M	0.61	1.78	23.00	7.20	132.00	28.30
22	F	0.97	1.84	10.00	11.20	178.00	45.80
28	M	1.40	3.70	14.00	4.00	97.00	14.10

### Leicester cough questionnaire (LCQ)

Patients were asked to report the severity of their symptom using LCQ pre- and post-operatively. LCQ results were calculated by the sum of scores from each of its three domains. Total LCQ score improved significantly from 11.9 (± 3.2) to 18.3 (± 1.2), *P *= 0.01 (CI: 2.2 to 10.4).

**Figure 1:**
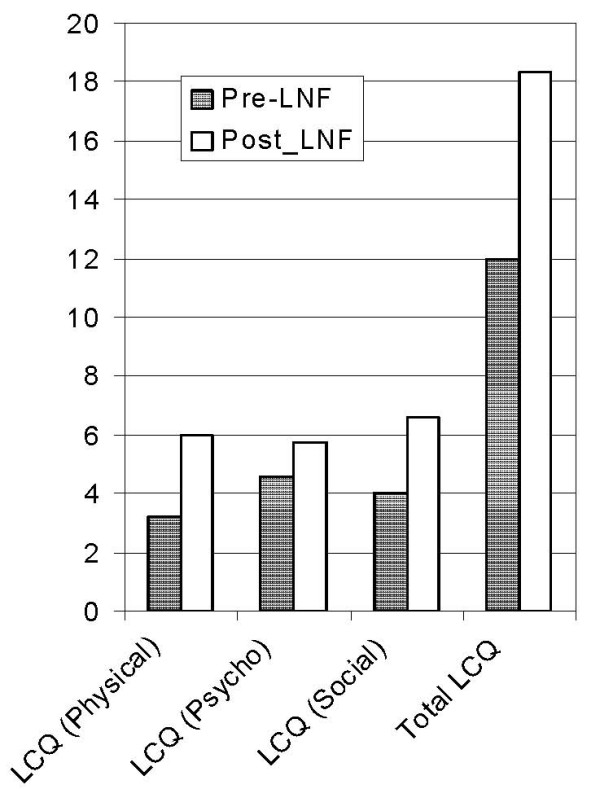
**LCQ domains before and after fundoplication**.

In the physical domain mean score increased from 3.2 (± 0.95) to 6(± 0.4), *P *= 0.002 (CI: 1.5 to 3.9). Mean scores from social domain increased from 4 (± 1.4) to 6.5 (± 0.6), *P *= 0.01 (CI: 0.8 to 4.2). In the psychological domain, scores increased from 4.5 (± 0.9) to 5.7(± 0.5), *P *= 0.06 (CI of -0.1 to 2.4). (Figure 1)

### Spirometry

Forced expiratory volume in one second (FEV1) increased from a mean of 1.03L (± 0.32) to 1.17L (± 0.41), *P *= 0.04 (95%CI = 0 to 0.25), and forced vital capacity (FVC) improved from a mean of 2.62L (± 0.88) to 2.87L (± 0.96) and *P *= 0.05.

### Exacerbation

**Figure 2:**
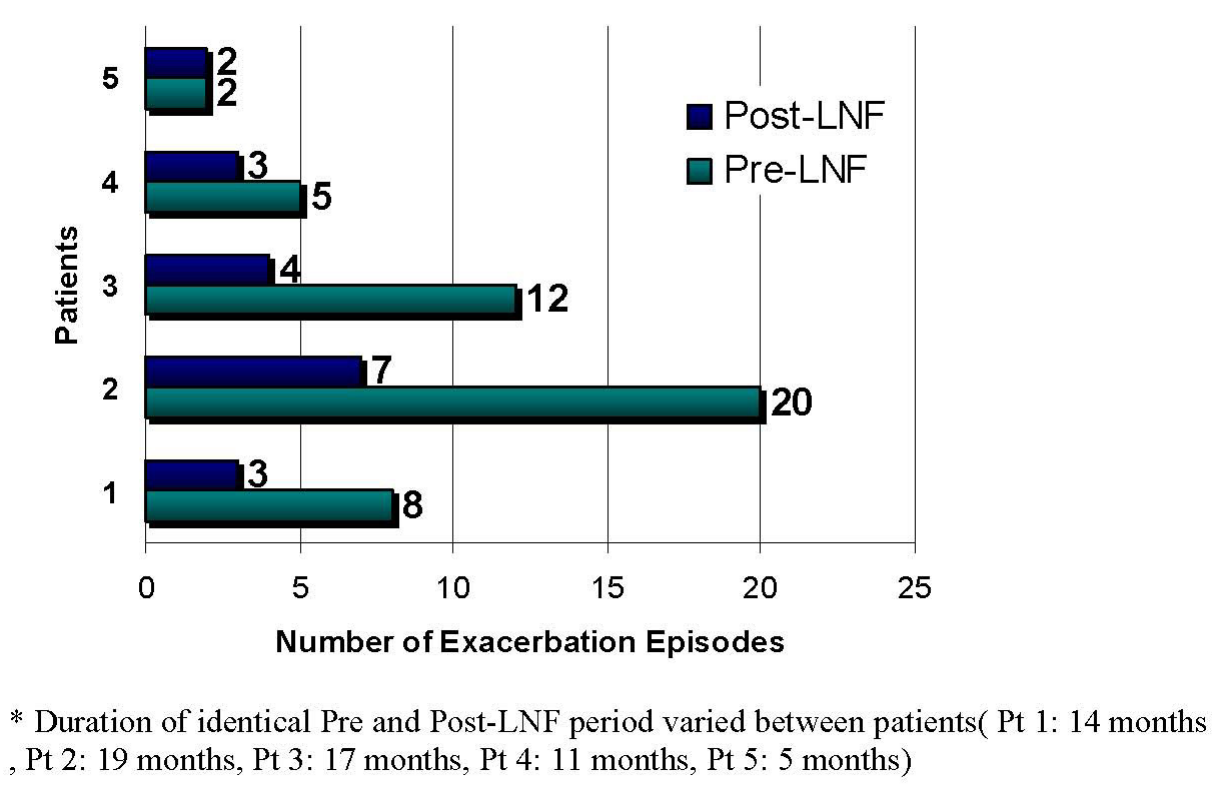
**Number of exacerbations in individual patients**.

Post fundoplication epoch was compared to the same pre-operation era for the number of exacerbations; which were defined as the need for oral or IV antibiotic therapy. The mean number of exacerbations halved from 8 (± 7.1) to 3.1 (± 2.3). In other words, patients had one exacerbation per two months before the operation, while it was one in 4 months after fundoplication (*P *= 0.01) Figure 2

## Discussion

Cough is the commonest symptom of medical importance. Chronic cough is a frequent symptom in general population with the incidence of 12%.[13] Whilst airway disease, particularly when characterized by eosinophilic inflammation, can commonly cause cough, gastroesophageal reflux is increasingly recognised as an important precipitant of coughing paroxysms. We have recently described characteristic features in the clinical history which point to reflux as a cause of cough.[8] Associations such as cough on eating, and post-prandially, cough precipitated by a change of posture or on rising in the morning, can be explained by the known physiology of the lower oesophageal sphincter. Extra-oesophageal manifestations such as dysphonia, and an unpleasant taste in the mouth are also important clinical pointers to reflux as a cause of coughing.

Cough is one of the characteristic symptoms of CF, initially presenting as pulmonary exacerbations. In a study of objective cough frequency in 14 CF children with exacerbations, cough rates per hour were reported as 18.2 during the day and 5.8 during the night. [14] Another study of 20 CF adults admitted with exacerbations demonstrated rates of 21.2 during the day and 4.8 at night.[15]

In some patients with CF, cough becomes a persistent chronic symptom. Patients will describe two sorts of cough. One productive and associated with exacerbations and a second dry cough usually characterised by irritation in the throat. We sought the clinical history of reflux cough in our patients with chronic cough and were struck by the high incidence of reflux associated features. As a result, we invited them to undergo oesophageal function studies.

In the normal western population, excessive acid reflux is a common finding with the prevalence of between 10 to 20%.[16] It is well recognised that CF patients have excessive incidence of acid reflux leading to high frequency of the classic symptoms of heart burn and dyspepsia.[17] A recent study has shown that CF patients with demonstrable reflux have a high incidence of increased bile acids in saliva, and they have definitive evidence of gastric aspiration, confirmed by the presence of bile acids in bronchoalveolar lavage fluid.[18] Moreover, significant reductions in FEV1 and FVC,[19] and progression of pulmonary disease due to reflux have been reported previously.[20]

Suggested mechanisms of excessive reflux included abnormalities of pancreatic and duodenal function leading to increased enteroglucagon levels, and hence delayed gastric emptying. Gastric acid secretion may be excessive.[21] Reflux may also be facilitated by oesophageal dysmotility, the head-down position during respiratory treatment or certain respiratory medications (e.g. theophylline).[22] The mechanical disruption of the lower oesophageal sphincter as a result of alterations in the shape of chest wall and flattening of the diaphragm in chronic lung disease can also precipitate reflux.

In all of our studied patients acid reflux was confirmed. The recommended strategy in treatment of reflux cough is therapeutic trials;[23] starting with high dose proton pump inhibitors plus ranitidine[24] (so called full acid suppression), to pro-motility agents (metoclopramide or domperidone) and cough suppressants such as low dose opioid.[25] On therapy, heart burn and dyspepsia were improved, but in contrast there was no or little change in the degree of the cough.

Fundoplication has been reported as extremely effective in the treatment and management of reflux symptoms.[26] It has been shown to be effective in the control of cough in patients with reflux (acid or non-acid), with or with out primary respiratory problems.[27,28] In our study, the beneficial effects of fundoplication in the form of reduced cough and improvement of voice were reported by the patients and noticed by the physician on the first post operative day. A formal assessment by LCQ of the longer term effects on quality of life showed a consistent benefit of the procedure. The increase in LCQ of 6 points in our patients is the highest clinically significant improvement reported, and nearly was double that seen from the effect of opiates in chronic cough.[25] The changes in spirometrically assessed lung function were small, and while of borderline statistically significance probably do not represent clinically important benefit. What was surprising was the marked reduction in exacerbation rate as defined by the need for antibiotic therapy. This is a non-controlled study and therefore regression to the mean or a placebo effect can not be discounted, however the halving of the incidence may suggest an important affect of fundoplication on pulmonary exacerbations. It is possible that reflux and aspiration maybe a precipitant or even a causal event in the pathogenesis of some pulmonary exacerbations. In support of this hypothesis, pH of exhaled breath condensate has been shown to be markedly lower in the acute phase of the exacerbations.[29]

Whilst the effect of fundoplication in CF has not previously been studied, experiences in the context of lung transplantation have shown improvement in episodes of acute and chronic rejection and the serial lung function.[30,31] These observations are again consistent with reflux and aspiration as a feature of pulmonary exacerbations, and have an important bearing in the context of CF, since many patients may progress to transplant assessment.

Technically, fundoplication is subtly more difficult in CF patients than in normal subjects with reflux. The tissues are generally more friable and edematous perhaps as a result of previous use of systemic steroids or as part of the systemic disease. In our patients, there were no operative or post-operative complications. Weight loss and dysphagia, recognised operative complications were trivial and short lived.

Our experience in the diagnosis and treatment of chronic cough, led us to the realisation that many patients with CF complain of a similar symptom profile indicative of reflux. In common with other reports, these patients have excessive acid reflux which we found to be unresponsive, in terms of cough, to medical anti-reflux therapy. Fundoplication produced a dramatic symptomatic improvement associated with a halving in the rate of pulmonary exacerbations. If this experience is replicated in larger studies, then reflux and aspiration may be demonstrated to be an important previously unrecognised mechanism of disease progression in CF.

## Competing interests

The authors declare that they have no competing interests.

## Authors' contributions

HF carried out data collection, literature review, statistical analysis, and drafted the manuscript. TM carried out data collection on lung functions and looked after the patients during exacerbations. JD looked after patients' diet. WJ carried out the 24 hour pH studies and manometries. PS carried out the operations and post op care. AHM conceived of the study, and participated in its design and coordination. All authors read and approved the final manuscript.

## Appendix 1

**Table:** Key points in history indicating the reflux origin of the cough

Hoarseness or a problem with the voice
Clearing the throat
Excess mucus in the throat, or drip down the back of the nose
Retching or vomiting when coughing
Cough on first lying down or bending over
Chest tightness or wheeze when coughing
Heartburn, indigestion, stomach acid coming up
A tickle in the throat, or a lump in the throat
Cough with eating (during or straight after meals)
Cough with certain foods
Cough when getting out of bed in the morning
Cough brought on by singing or speaking (for example, on the telephone)
Coughing during the day rather than night
A strange taste in the mouth
